# 

**Published:** 1904-07-30

**Authors:** 


### NOTICE TO CORRESPONDENTS.

All MSS., Letters, Books for Review, and other matters intended for the Editor should be addressed The Editor, "The Hospital," The Hospital Buildings, 28 & 29 Southampton Street, Strand, W.C.

The Editor cannot undertake to return rejected MS., even when accompanied by stamped directed envelope.

**Notice to Advertisers and Subscribers.**

All Advertisements, Orders for Copies of The Hospital, and Business Communications should be addressed to **THE MANAGER (Not to the Editor),** The Hospital, 28 & 29 Southampton Street, Strand, London, W.C.

The Cost of Subscription, post free, is as follows:—Per week, 3½d.; per quarter, 3s. 3d.; per half-year, 6s. 6d.; per annum, 13s. Foreign and Colonial:—Per annum, 19s. 6d., payable, in all cases, in advance.

**NOTICE.—Vol. XXXV. of "The Hospital" (October 1903 to March 1904), in handsome cloth binding, is now on sale at the office of this Journal, price 10s. 6d. Cases for binding the Numbers contained in this Volume 2s. Index to Vol. XXXV., 3½d., post free.**

**The Monthly part for August will be ready on August 1. Price 1s., by post, 1s. 3d.**

### BOOKS RECEIVED.

Kegan Paul, Trench Trübner and Co.
"Childhood in Health and Sickness."

Reynolds and Branson.
"Emergency Pocket Case-Book."

W. B. Saunders and Co.
"Obstetric and Gynæcologic Nursing" By Edward P. Davis.

E. Marlborough and Co.
"Dutch Self-Taught." By Captain C. A. Thimm.

W. H. and L. Collingridge.
"Easily Grown Hardy Perennials." By George H. Vos, M.B., B.A., etc.

Scientific Press, Limited.
"The Pocket Book of Temperature Charts."

Baillière, Tindall and Cox.
Medical Monograph Series: "Adenoids."
"Practical Medical Electricity."
"Diseases of the Stomach."
"The House of Chalie, 1700-1900."

Eliot Stock.
"Municipal Shortcomings."

Thomas Nelson and Sons.
"The Romance and Realm of Commerce." By Alfred Morris.

Walter Hill.
"Seaside, Farmhouse, Country Lodging and Hotel Guides."
"The Holidays, 1904: Where to Stay and What to See."

### Medical Appointments.

**R. C. Bartlett**, M.R.C.S., L.R.C.P., Certifying Factory Surgeon for the Romsey District, Hants. **E. H. Boulby**, M.D., M.R.C.S, L.R.C.P., Clinical Assistant to the Chelsea Hospital for Women. **F. R. Eddison**, M.R.C.S., L.R.C.P., Medical Officer of Health, Bedale Rural District. **J. Comyns Leach**, M.D., B.Sc., D.P.H.Camb. and Lond., reappointed permanently Medical Officer of Health for the Sturminster District. **F. H. McCaughey**, M.B., B.S.R.U.I., Resident Assistant Medical Officer for the Bradford Union. **T. T. Macklin**, M.D., Medical Officer of Health, Clitheroe Rural District. **C. G. Meade**, L.S.A., District Medical Officer of the Southmolton Union. **Percy Lockhart Mummery**, B.C.Cantab. F.R.C.S.Eng., Assistant Surgeon to the North-Eastern Hospital for Children, Hackney Road. **F. C. Neal**, M.R.C.S., L.R.C.P., Clinical Assistant, Chelsea Hospital for Women. **H. G. Pesel**, M.D., B.S.Edin., Resident Assistant Medical Officer at the Eastby Sanatorium of the Bradford Union. **J. P. Philip**, M.D.Aberd.. D.P.H., Medical Officer of Health, Morpeth Rural District. **J. Houston Porter**, M.R.C.S., L.R.C.P., House-Physician to the London Hospital. **William Sankey**, M.B., Ch.B.Vict. Surgeon for the B Division of the Manchester City Police. **H. J. Walker**, M.R.C.S., L.R.C.P., Certifying Factory Surgeon for the Brighton District, Sussex. **A. Walter Williams**, M.R.C.S., L.S.A., Surgeon for the A Division of the Manchester City Police.

**"The Hospital" Institutional Library.**

**"Hospitals and Asylums of the World." 4 Vols., with a Portfolio of Plans, complete £12 12s.**

**"Burdett's Hospitals and Charities, 1904." 5s. net.**

**"Burdett's Official Nursing Directory." 8s. net.**

**"Cottage Hospitals, General, Fever, and Convalescent." 10s. 6d.**

**"Uniform System of Accounts for Hospitals and Public Institutions." New Edition. 4s. net.**

**"Hospital Expenditure: The Commissariat." 2s. 6d.**

**"A Legal Handbook for the Use of Hospital Authorities." 1s. 6d.**

**"The Cottage Hospital Case Book or Register of Patients." 10s. and 12s. 6d.**

**All these are published by the Scientific Press, Ltd., and may be obtained through any bookseller, or direct from the publishers, 28 and 29 Southampton Street, Strand, London, W.C.**

### MANUALS FOR NURSES.

### GENERAL NURSING.

**A HANDBOOK FOR NURSES.** 5/- net, 5/5 post free.
By J. K. WATSON, M.D.
New and Revised Edition. Profusely illustrated.

**NURSING: ITS THEORY AND PRACTICE.** 3/6 post free.
By PERCY G. LEWIS, M.D.
A Complete Text-Book of Medical, Surgical, and Monthly Nursing.

**NURSING: ITS PRINCIPLES AND PRACTICE.** 7/6 net, 7/10 post free.
By ISABEL ADAMS HAMPTON.
New and Enlarged Edition. Illustrated.

**THE NURSES' DICTIONARY OF MEDICAL TERMS AND NURSING TREATMENT.** 2/- post free.

Compiled by HONNOR MORTEN.

### MIDWIFERY.

**A COMPLETE TEXT-BOOK OF MIDWIFERY.** 6/- net, 6/3 post free.
By J. K. WATSON, M.D.
Over 350 pp., profusely illustrated, with 143 Diagrams.

**A PRACTICAL HANDBOOK OF MIDWIFERY.** 6/- net, 6/3 post free.

By FRANCIS W. HAULTAIN, M.D.

New and Revised Edition. Profusely illustrated.

**THE MIDWIVES' POCKET BOOK.** 1/- post free.

By HONNOR MORTEN.

**HOW TO BECOME A CERTIFIED MIDWIFE.** 1/- net, 1/1 post free.
By E. L. CAPPEL, M.B., B.S., B.Sc. Lond.
Contains all regulations for the Midwife and for Local Authorities.

### SPECIAL TEXT-BOOKS.

**NURSING IN DISEASES OF THE THROAT, NOSE, AND EAR.** 2/6 post free.

By P. MACLEOD YEARSLEY, F.R.C.S.

**OPHTHALMIC NURSING.** 3/6 net, 3/10 post free.

By SYDNEY STEPHENSON, M.B.

New and Revised Edition. Illustrated.

**SURGICAL WARD WORK AND NURSING.** 3/6 net, 3/10 post free.

By ALEXANDER MILES, M.D.

Second Edition. Illustrated.

**GYNÆCOLOGICAL NURSING.** 1/- post free.
By G. A. HAWKINS-AMBLER, F.R.C S.

### BANDAGING & MASSAGE.

**PRACTICAL GUIDE TO SURGICAL BANDAGING AND DRESSINGS.** 2/- post free.

By WM. JOHNSON SMITH, F.R.C.S.

Profusely Illustrated.

**ART OF MASSAGE.** 6/- post free.
By Mrs. CREIGHTON HALE.
Second Edition. Profusely Illustrated.

**MEDICAL GYMNASTICS.** 2,6 post free.
Including the Schott (Naubeim) Movements. Being a Text-Book of Massage and Mechanical Therapeutics Generally. Illustrated.
By AXEL V. GRAFSTROM, B.Sc., M.D.

Complete Catalogue Post Free on Application.

*The above Works can be obtained of all Booksellers, or of*
THE SCIENTIFIC PRESS, LTD., 28 & 29 Southampton St., Strand, London, W.C.

